# Calcium signaling controls early stage biofilm formation and dispersal in *Vibrio fischeri*

**DOI:** 10.1128/jb.00077-25

**Published:** 2025-05-14

**Authors:** Jeremy J. Esin, Karen L. Visick, Abby R. Kroken

**Affiliations:** 1 Department of Microbiology and Immunology, Loyola University Chicago 2456, Maywood, Illinois, USA; University of Illinois Chicago Pharmaceutical Sciences, Chicago, Illinois, USA

**Keywords:** video microscopy, *Vibrio fischeri*, biofilms, dispersal, quorum sensing

## Abstract

Bacterial dispersal from a biofilm is presently the least-studied step of the biofilm life cycle. The symbiotic bacterial species *Vibrio fischeri* is a model organism for studying biofilms relevant to a eukaryotic host; however, methodology is lacking to readily study the dispersal of this microbe from biofilms formed in the lab. Here, we adapted a time-lapse assay to visualize biofilm dispersal by *V. fischeri*. We observed biofilm formation and dispersal for multiple *V. fischeri* isolates, which displayed a variety of biofilm architecture phenotypes and dispersal dynamics. We then investigated *V. fischeri* strain ES114 using genetic tools and mutants available for this strain. ES114 exhibited calcium-dependent biofilm formation followed by a rapid (less than 10 min) coordinated dispersal event that occurred approximately 5 h from the experimental start. Biofilm dispersal was largely independent of the dispersal-promoting protease encoded by *lapG*. Although we found no role under our conditions for either biofilm formation or dispersal for several other factors including polysaccharides and autoinducers, we determined that biofilm formation was enhanced, and dispersal was delayed, with increased concentrations of calcium. Furthermore, biofilm formation depended on the calcium-responsive diguanylate cyclase (DGC) CasA, and dispersal could be modulated by overexpressing CasA. Our work has thus developed a new tool for the *V. fischeri* field and uncovered a key role for calcium signaling and c-di-GMP in early biofilm formation and dispersal in *V. fischeri*.

**IMPORTANCE:**

Biofilm formation and dispersal are critical steps in both symbiotic and pathogenic colonization. Relative to biofilm formation, the process of dispersal in the model symbiont *Vibrio fischeri*, and other bacteria, is understudied. Here, we adapted an imaging assay to study early biofilm formation and the dispersal process in *V. fischeri*. We demonstrated that our assay can quantify biofilm formation and dispersal over time, can reveal phenotypic differences in diverse natural wild-type isolates, and is sensitive enough to investigate the impact of environmental factors. Our data confirm that calcium is a potent biofilm formation signal and identify the diguanylate cyclase CasA as a key regulator. This work leads the way for more in-depth research about unknown mechanisms of biofilm dispersal.

## INTRODUCTION

Biofilm formation is a survival strategy of pathogenic, symbiotic, and environmental bacterial species (1). Biofilms can provide protection from environmental stressors such as antibiotics, host immune-mediated damage, and shear forces (2). Importantly, they can also serve as a reservoir for subsequent infection elsewhere through dispersal, a regulated process that can occur in response to a variety of signals, including starvation and environmental cues (3, 4). Although genetic and environmental factors that promote biofilm formation have been rigorously studied in many microbes, the study of biofilm dispersal can be more challenging to observe and measure. Due to technological advances in microscopy and image analysis and expanded knowledge of the biofilm formation process, the study of biofilm dispersal has become more prevalent in recent years.

The marine bacterium *Vibrio fischeri* has been extensively studied for biofilm formation as it forms and disperses from biofilms to colonize its host, the Hawaiian bobtail squid *Euprymna scolopes* (5). These studies have identified three major pathways critical for the formation of the complex *V. fischeri* biofilm (reviewed in [5]). Two polysaccharides, SYP (symbiosis polysaccharide) and cellulose, and a large adhesive protein, LapV, have been shown to contribute to biofilm formation *in vitro* (6–8). SYP, produced by proteins encoded by the *syp* locus, is best known for its critical role in promoting host-associated biofilm formation and colonization (e.g., [6, 9–11]). SYP fosters cell-cell aggregation and imparts cohesive properties in the laboratory setting (10). However, SYP production is minimal under standard laboratory conditions by the most commonly used wild-type strain, ES114, necessitating the use of genetically manipulated strains for its study. In such strains, it has been shown that biofilm formation can be substantially induced by the introduction of calcium, but not other cations (e.g., [12]).

In contrast to SYP’s cell-cell aggregation function, cellulose promotes cell-surface attachment in liquid cultures, for example, to the glass test tube in shaking liquid cultures (9, 10, 12, 13). It also contributes, to a lesser extent, to colony morphology by enhancing wrinkling architecture. Cellulose-dependent phenotypes are also induced by calcium supplementation, particularly at levels of 10 mM (which approximates that present in seawater) and higher (8, 12, 14). This effect occurs in part at the level of transcription of the cellulose genes, *bcs*. Induction of *bcs* transcription by calcium requires the diguanylate cyclase (DGC) CasA and transcription factor VpsR (14).

DGCs function by synthesizing c-di-GMP (Bis-(3’,5’)-cyclic dimeric guanosine monophosphate), a molecule that promotes biofilm formation in many bacteria (5, 15, 16). In turn, c-di-GMP is degraded by phosphodiesterases (PDEs). Many bacterial species encode multiple DGCs and PDEs to fine-tune c-di-GMP levels in response to environmental signals (15). In *V. fischeri*, CasA responds to calcium by synthesizing c-di-GMP via its cytoplasmic GGDEF enzymatic domain (14). Disruption of *casA* prevents both the increase in c-di-GMP levels in response to calcium addition and the associated calcium-dependent phenotypes. These defects are complemented by a wild-type copy of *casA* but not by a mutant derivative with a change in the conserved GGDEF motif (G410A), demonstrating that the calcium-induced changes were caused by CasA-produced c-di-GMP.

Low concentrations of c-di-GMP can promote dispersal from biofilms. For example, in *Pseudomonas fluorescens*, c-di-GMP controls the surface localization of a large adhesive protein, LapA, to mediate the switch between biofilm and dispersal (17–19). When c-di-GMP levels are low, the protease LapG cleaves LapA from the cell surface, promoting dispersal. In contrast, when levels are high, they bind to the inner membrane protein LapD, changing its conformation to permit it to sequester LapG, decreasing dispersal and promoting biofilm formation. In *V. fischeri,* a homologous c-di-GMP-controlled dispersal pathway exists that includes LapD and LapG, along with a non-homologous large adhesive protein, LapV (7). In *V. fischeri*, the deletion of *lapG* increases the proportion of bacteria associated with biofilms in shaking broth cultures, whereas *lapD* mutants exhibit diminished biofilm formation, presumably due to being “locked” into a dispersal state (7). LapV is hypothesized to bind to both SYP and cellulose exopolysaccharides and potentially to surfaces to facilitate both surface attachment and cell-cell interactions in biofilms. Beyond this pathway, however, nothing is known about dispersal in *V. fischeri*.

In this study, we adapted a time-lapse microscopy method developed by Bridges and Bassler (20) to visualize the biofilm formation and dispersal dynamics of *V. fischeri*. We observed different patterns of growth and dispersal for diverse strains of *V. fischeri*. For the best-studied strain, ES114, we observed a coordinated dispersal event that was independent of known autoinducers. Unexpectedly, early biofilm formation did not require SYP, cellulose, or the LapV surface adhesin. However, calcium was critical for bacterial attachment and accumulation of biofilms. Finally, we identified an important role for the calcium-sensing DGC CasA. Together, these data reveal new biofilm formation and dispersal dynamics as well as describe a key regulatory factor important for these processes in *V. fischeri*.

## RESULTS

### Squid- and fish-derived isolates of *V. fischeri* exhibit diverse aggregation and dispersal phenotypes

To explore how *V. fischeri* forms and, more importantly, disperses from a biofilm, we adapted the static time-lapse microscopy strategy originally described for *V. cholerae* by Bridges and Bassler (20). Briefly, bacterial samples were diluted, plated into an 8-well borosilicate glass dish, and allowed to attach for 1 h, after which the wells were washed and overlaid with fresh medium prior to imaging. Fields of view containing small groups of attached bacteria, or multiple single cells, were chosen and then imaged every 5 min over 10 h to observe the entire biofilm formation and dispersal lifecycle. We performed these assays using the Tris-buffered tryptone medium tTBS (which supports biofilm formation when calcium is added [21]), in chambers held at 25°C.

**Fig 1:**
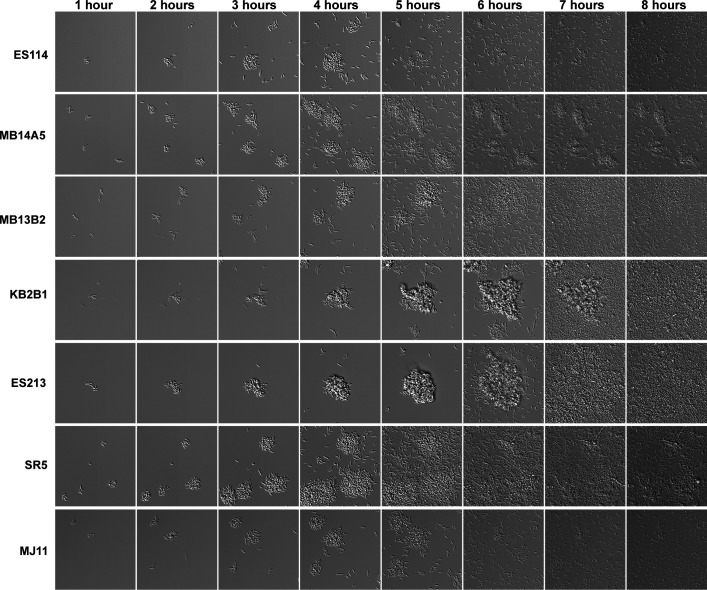
Wild-type squid and fish isolates have diverse biofilm phenotypes. Representative differential interference contrast microscopy (DIC) images from 1–8 h of a 10-h experiment of various wild-type *V. fischeri* isolates. Experiment performed in tTBS containing 10 mM CaCl_2_ from the point of plate inoculation in room temperature chambers (25°C). Images displayed are 91 µm wide. The images are representative of *n* = 3 biological replicates. The low-contrast vertical banding in images is an artifact from the DS-Qi2 CMOS camera.

ES114 is the most widely studied *V. fischeri* squid isolate, particularly with respect to biofilm formation, but other isolates also obtained from wild-caught squid and fish hosts are studied in laboratory settings (22–27). We evaluated a set of seven isolates for attachment, biofilm size, and dispersal patterns during growth in tTBS containing 10 mM calcium. Strains ES114, MB14A5, MB13B2, KB2B1, ES213, SR5, and MJ11 all attached and formed biofilms, but with some distinct phenotypes (Fig. 1; Movie S1). Strain ES114 attached to the glass bottom, multiplied, formed small biofilms, and then dispersed in a rapid and coordinated manner between 4 and 5 h. ES213 and KB2B1 formed larger aggregates that appeared to disperse later than ES114 (e.g., between 6 and 8 h). MB14A5 and SR5 did not disperse completely in the timeframe of the experiment, whereas MB13B2 and MJ11 formed aggregates with a size similar to ES114 but had delayed dispersal timing. The strains displayed different biofilm formation timing, maximum sizes, and dispersal phenotypes, indicating that different strains of *V. fischeri* recognize and respond in distinct ways to the conditions of our assay. We anticipate that further exploration of these varied isolates will provide additional insights into the mechanisms underlying biofilm formation and coordinated dispersal. Here, we pursued the investigation of dispersal factors in ES114 due to both the larger number of tools available for this strain and the fact that it disperses when the numbers of planktonic individuals are relatively low, making the dispersal event more accessible for imaging and quantification.

### Attachment and early aggregation do not depend on SYP, cellulose, or LapV

**Fig 2:**
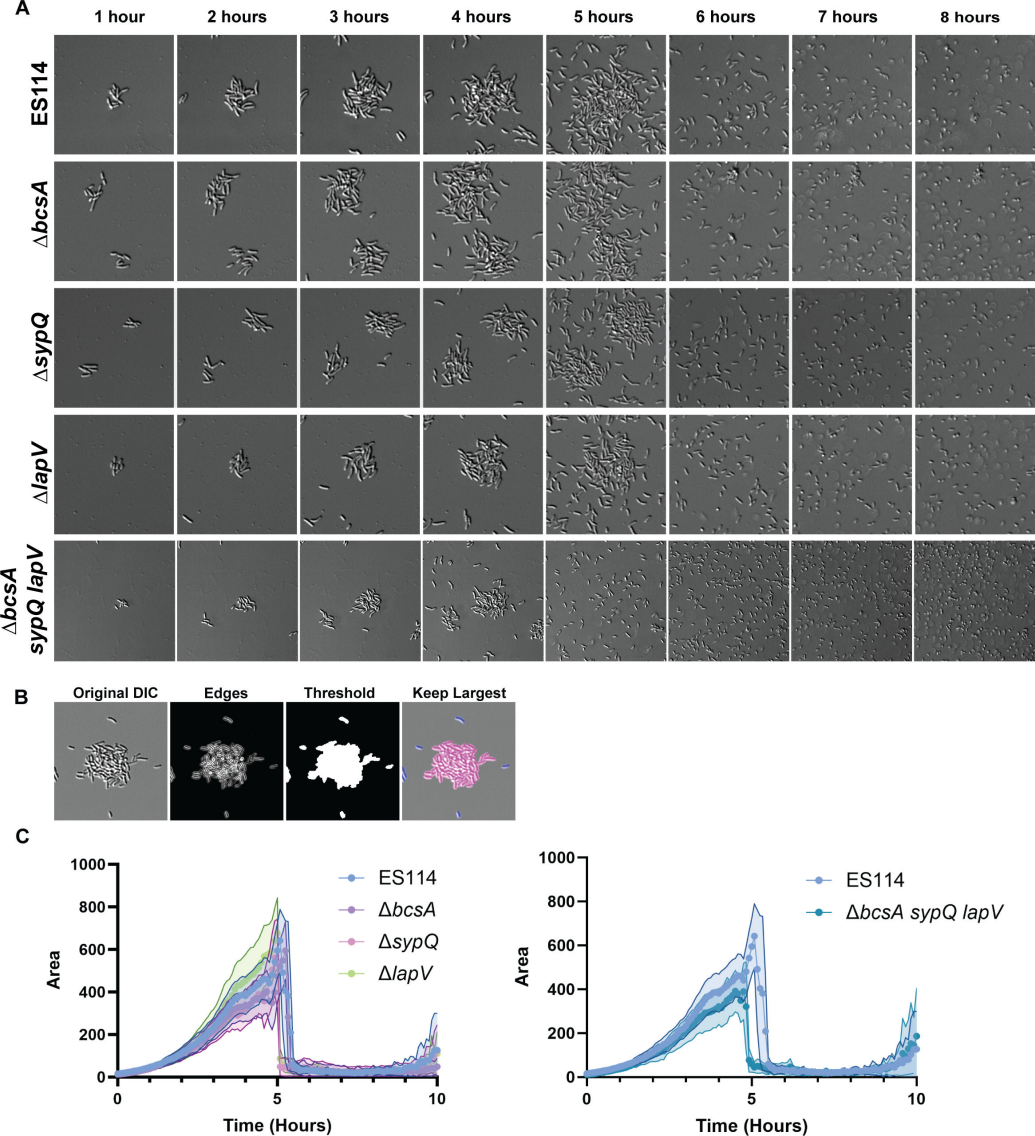
Early aggregation and attachment are independent of SYP, cellulose, and LapV. (**A**) Representative DIC images from 1–8 h of a 10-h experiment performed as in Fig. 1. (**B**) Schematic of steps taken in the ImageJ macro used to quantify biofilm area. See text for details. (**C**) Quantification of biofilm area as measured by time-lapse microscopy from a representative experiment for ES114 and mutants defective for SYP (∆*sypQ*), cellulose (∆*bcsA*), or LapV surface adhesin (∆*lapV-1500*), or a triple mutant deficient for all 3 (∆*bcsA* Δ*sypQ* Δ*lapV-1500*). Images displayed are 46 µm wide. The images are representative of *n* = 3 biological replicates, and the quantifications include *n* = 15–25 biofilms per condition, ± Standard deviation (SD) (Shaded).

Considering that biofilm dispersal first requires bacteria to form a biofilm, we tested whether known *V. fischeri* biofilm components were required for attachment, early aggregation, or biofilm formation in our assay. Single mutants with disruptions in genes required for the production of SYP (Δ*sypQ*), cellulose (Δ*bcsA*), and LapV (Δ*lapV*) were evaluated. Previous work from our lab showed that each of these components was at least partially involved in attachment to borosilicate glass during biofilm formation in shaking liquid (7, 12), and therefore, we hypothesized that they would be required here. However, each single mutant exhibited biofilm formation and dispersal phenotypes similar to ES114 (Fig. 2A; Movie S2). These data suggest that the loss of any one of these surface components is not sufficient to disrupt attachment, biofilm formation, or the dispersal process.

To permit subsequent comparisons between replicates, mutant strains, and other experimental conditions, we analyzed each image using a macro designed in the software ImageJ (FIJI) (28). In brief, the macro segments bacterial aggregates from both background and planktonic bacteria and tracks 2D area over time (see Materials and Methods for further details) (Fig. 2B). Fifteen to 25 individual biofilms were measured from each condition during each experimental replicate, and experiments were repeated two to three times. Since the absolute dispersal time varied between biological replicates (Fig. S1), single representative replicates are shown, and additional replicates are available in supplementary figures. This quantification confirmed that no single surface component deletion was sufficient to alter the pattern of dispersal (Fig. 2C; Movie S2). We thus asked if two or more of these known biofilm factors were necessary by evaluating a triple *bcsA sypQ lapV* mutant that is defective for all three biofilm components. Like the single mutants, the triple mutant also attached, formed a biofilm, and dispersed during time-lapse experiments (Fig. 2A and D; Fig. S2B; Movie S2), although the mutant dispersed from the biofilm 5–20 min earlier than ES114 in each replicate. These data suggest other factor(s) contribute to attachment and steps leading up to early biofilm formation.

### *V. fischeri* dispersal from early biofilms is largely independent of *lapG*

**Fig 3:**
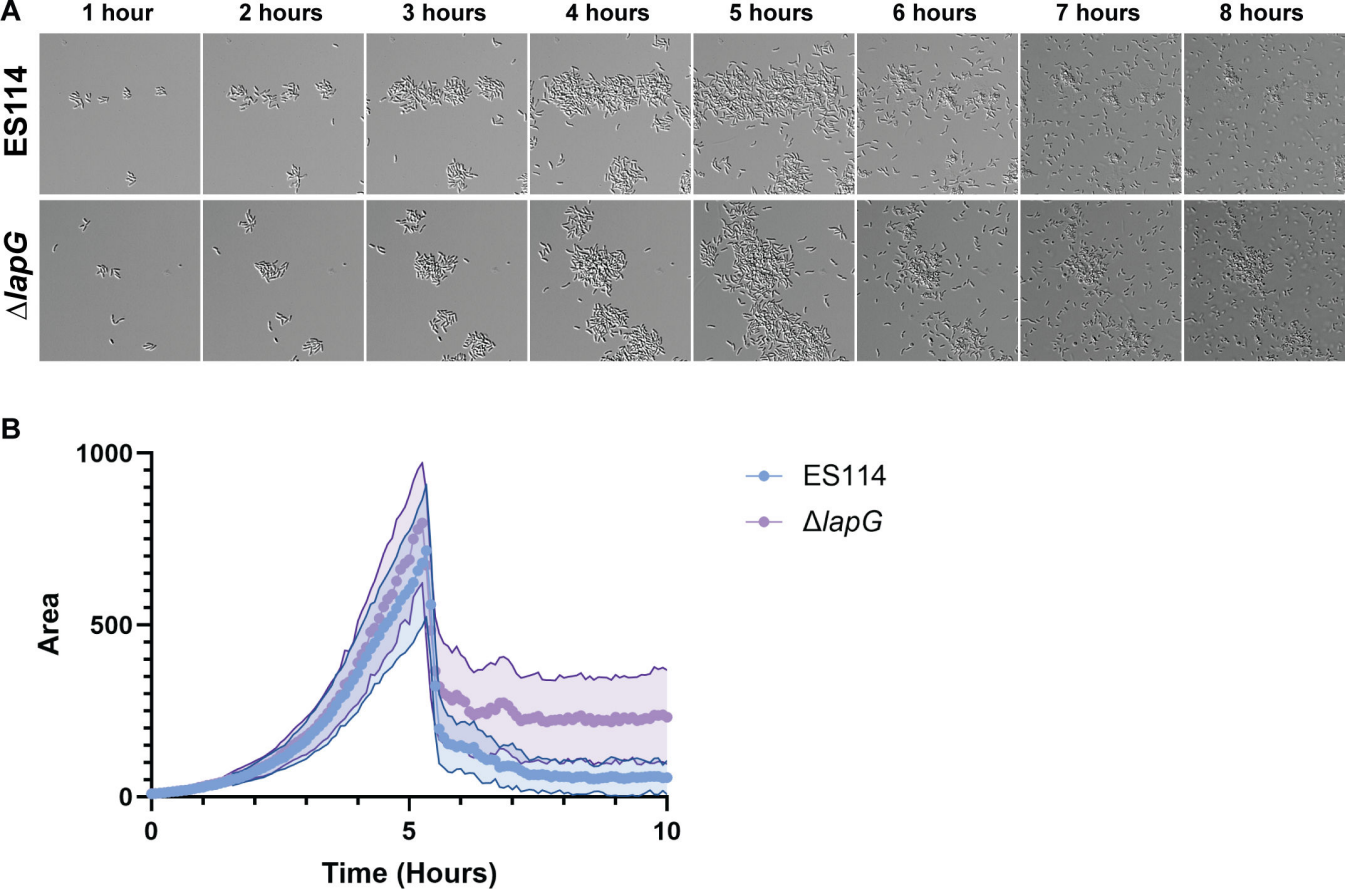
*V. fischeri* dispersal from early biofilms is largely independent of *lap*G. (**A**) Representative DIC images of 1–8 h of a 10 h experiment, performed as in Fig. 1, comparing ES114 with a strain deficient for LapG (∆*lapG*). (**B**) Quantification from a representative experiment via ImageJ macro. Images displayed are 46 µm wide. The images are representative of *n* = 3 biological replicates, and the quantifications include *n* = 15–25 biofilms per condition, ± SD (Shaded).

To evaluate mechanisms of biofilm formation and dispersal under conditions that promote ES114 biofilm formation, we tested a *lapG* protease mutant. LapG-mediated cleavage of the surface adhesin LapV promotes dispersal (7), and thus, we hypothesized that the *lapG* mutant would be incapable of dispersal. Surprisingly, the *lapG* mutant was competent to disperse (Fig. 3A; Movie S3). Quantification revealed that the majority of the *lapG* mutant cells dispersed in a fashion similar to ES114 (Fig. 3B; Fig. S3). Of note, small areas of biofilm remain attached past the dispersal event, an observation that was consistent across our replicates (Fig. S3). These areas were larger in the *lapG* mutant condition (Fig. 3B, 6–10 h), suggesting that the mutant has a slight defect in biofilm dispersal. The ability of the *lapG* mutant to attach and initiate dispersal suggests that *V. fischeri* uses another pathway(s) to control early biofilm formation and dispersal.

### Coordinated dispersal is independent of autoinducer signaling

**Fig 4:**
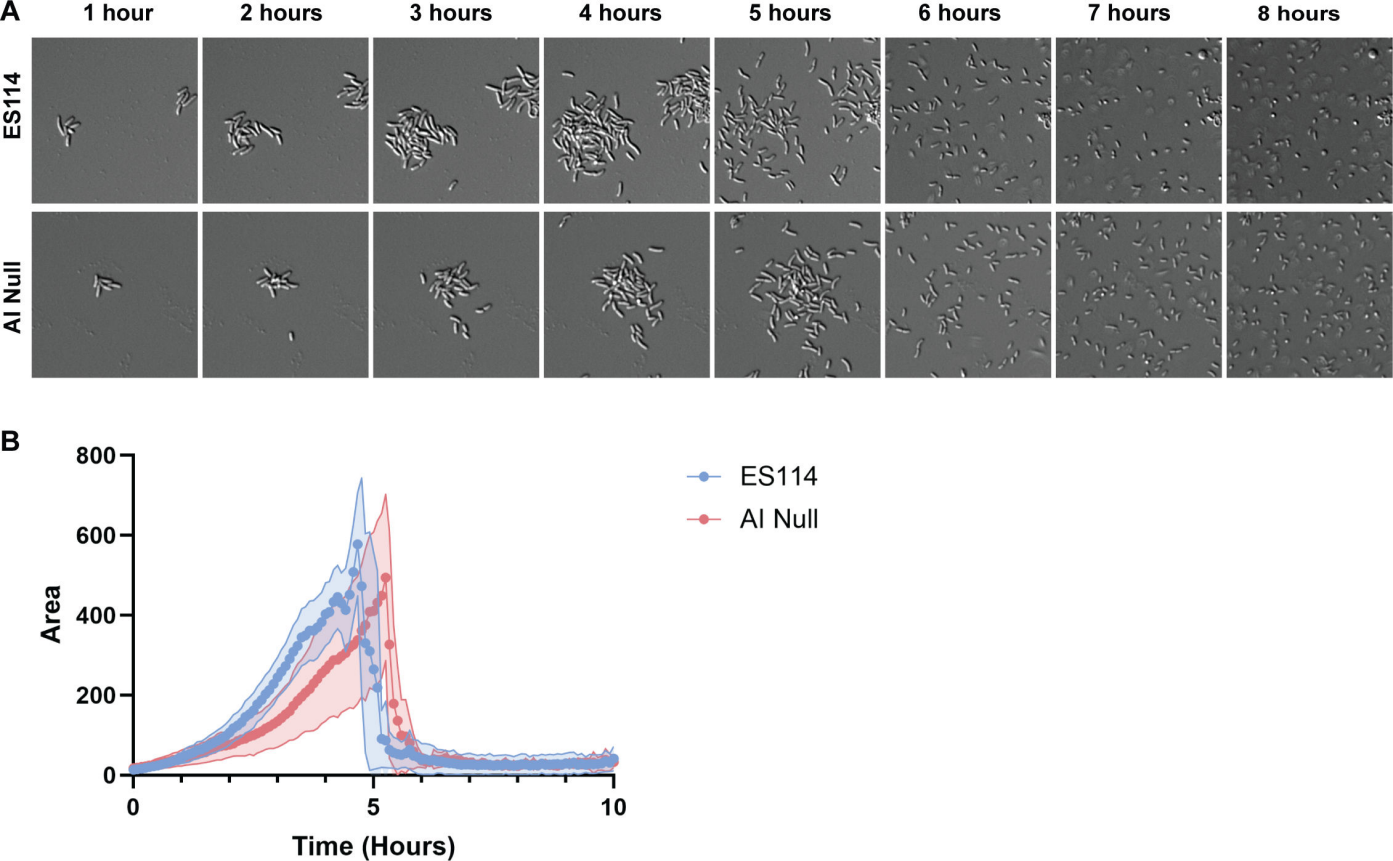
Coordinated dispersal is independent of autoinducer signaling. (**A**) Representative DIC images of 1–8 h of a 10 h experiment, performed as in Fig. 1, comparing ES114 with a strain deficient for all three autoinducers (*luxI*-frameshift ∆*ainS luxS*). (**B**) Quantification from a representative experiment via ImageJ macro. Images displayed are 46 µm wide. The images are representative of *n* = 3 biological replicates, and the quantifications include *n* = 15–25 biofilms per condition, ± SD (Shaded).

Given the rapid and coordinated dispersal that we observed (Fig. 2), we postulated that the production and accumulation of autoinducers (29, 30) functions to promote ES114 dispersal. To test this possibility, we evaluated biofilm dispersal by a strain that lacks the three known autoinducers normally produced by *V. fischeri* (i.e*.*, defective for *luxI*, *ainS*, and *luxS*). Contrary to our hypothesis, this triple mutant formed aggregates, achieved a similar overall aggregate area, and dispersed in a coordinated manner comparable with the wild-type parent. While there was some variance between replicates (Fig. 4; Fig. S4; Movie S4), there was no consistent trend toward earlier or later dispersal. These data suggest that autoinducers are not required for early dispersal but leave open the possibility that other secreted molecules could be involved.

### *V. fischeri* strain ES114 forms and disperses from biofilms when calcium is present

**Fig 5:**
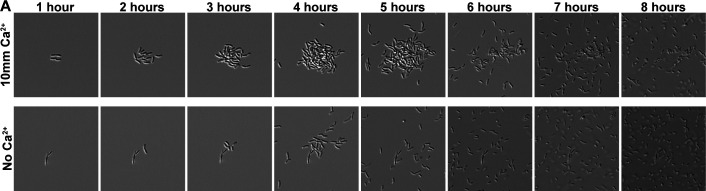
Calcium is required for aggregate formation. Representative DIC images of 1–8 h of a 10 h experiment comparing ES114 in tTBS media with 10 mM added calcium to ES114 in tTBS without added calcium. Other than the inclusion of media that lacks added calcium, the experiment was performed as in Fig. 1. Images displayed are 46 µm wide. *n* = 3 biological replicates.

Early biofilm formation and dispersal under our conditions were not impacted by autoinducer signaling, so we investigated environmental signals. Previous research has shown that calcium is a positive signal for biofilm formation in *V. fischeri* (12, 21, 31)*,* but it was unclear if calcium would play a role under the conditions of this new dispersal assay. Thus, we compared the ability of ES114 to form and disperse from biofilms in the presence and absence of 10 mM calcium chloride. When 10 mM calcium chloride was added to tTBS, ES114 readily formed and dispersed from biofilms, as seen previously (e.g., Fig. 1 and 2). In contrast, in non-supplemented tTBS conditions (lacking added calcium), ES114 did not form strong attachments to the glass slide, nor did the strain make multi-cell biofilms (Fig. 5; Movie S5). These data suggest that under our conditions, calcium is required to initiate the biofilm attachment and formation process.

### Increasing calcium delays biofilm dispersal

**Fig 6:**
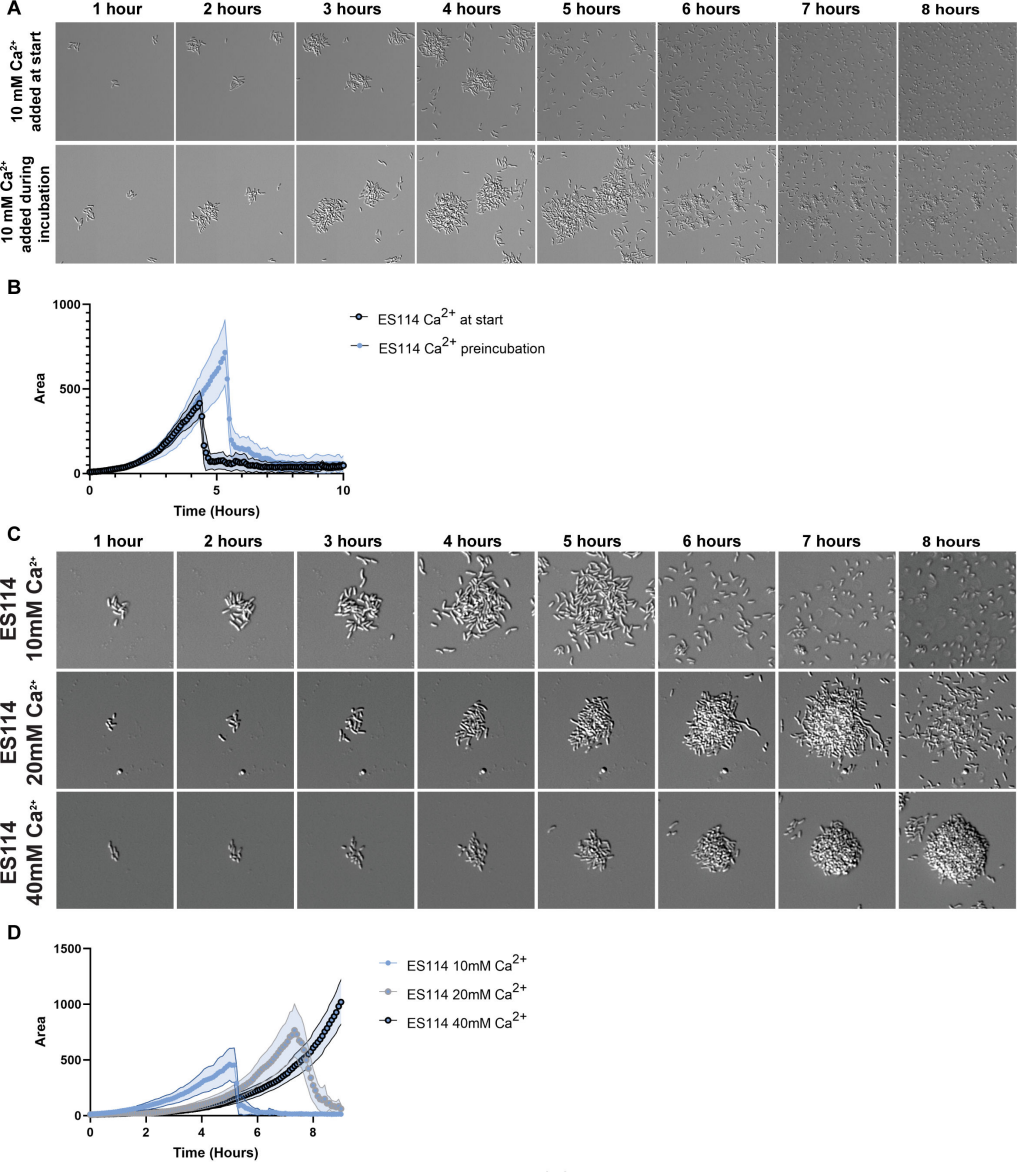
Calcium time of addition and concentration. (**A**) Representative DIC images of 1–8 h of a 10 h experiment comparing ES114 with 10 mM calcium added just prior to imaging and ES114 that was exposed to 10 mM calcium in the 1 h incubation period. (**B**) Quantification from a representative panel A experiment via ImageJ macro. (**C**) Representative DIC images of 1–8 h of a 10 h experiment comparing ES114 in three calcium concentrations (10 mM, 20 mM, and 40 mM). (**D**) Quantification of a representative experiment for panel C. Images displayed are 46 µm wide. The images are representative of *n* = 3 biological replicates, and the quantifications include *n* = 15–25 biofilms per condition, ± SD (Shaded). The low-contrast vertical banding in panel A is an artifact from the DS-Qi2 CMOS camera.

The requirement for calcium addition to induce cell attachment led us to hypothesize that the time of addition and/or concentrations of calcium in our assay could influence biofilm dispersal. First, we asked if the timing of calcium addition could change the outcome of the dispersal assay. The experimental setup includes a 1-h incubation to allow bacterial attachment. We thus compared whether including or excluding calcium during this incubation period changed biofilm formation or dispersal. When calcium was included during the 1-h incubation period, the biofilms formed by ES114 accumulated to occupy larger areas and dispersed later than those that were exposed to calcium after wash steps (Fig. 6A and B; Fig. S5A; Movie S6). These data demonstrate that *V. fischeri* is sensitive to the duration of calcium exposure, which when prolonged results in larger biofilms that disperse later.

Due to the significant effects of a change in the time of calcium addition, we tested if changing the concentration of calcium would alter biofilm formation and dispersal patterns. In our initial experiments, we used calcium at a concentration of 10 mM, which reflects the levels of calcium present in seawater. Previous research in *V. fischeri* demonstrated that calcium concentrations beyond 10 mM can impact other processes, such as motility and c-di-GMP levels (14, 32, 33). We therefore examined the impact of elevated calcium concentrations (20 mM and 40 mM) on biofilm formation and dispersal. Indeed, ES114 produced larger biofilms in response to higher amounts of calcium (Fig. 6C; Movie S6). Strikingly, the timing of dispersal was also delayed when higher amounts of calcium were present, as ES114 reached a maximal biofilm area at later time points (Fig. 6D; Fig. S5B). The appearance of the biofilms in 20 mM and especially in 40 mM calcium was also altered; both had a more rounded and compact structure compared with the 10 mM calcium condition, which consisted of a looser aggregate. The circular structure of ES114 in 40 mM calcium conditions more strongly resembled the biofilms made by ES213 and KB2B1 (compare Fig. 6C with Fig. 1). Overall, these data indicate that calcium exposure, both duration and concentration, can influence biofilm formation, peak biomass, and time of dispersal for *V. fischeri*.

### Chelation of free calcium promotes biofilm dispersal

**Fig 7:**
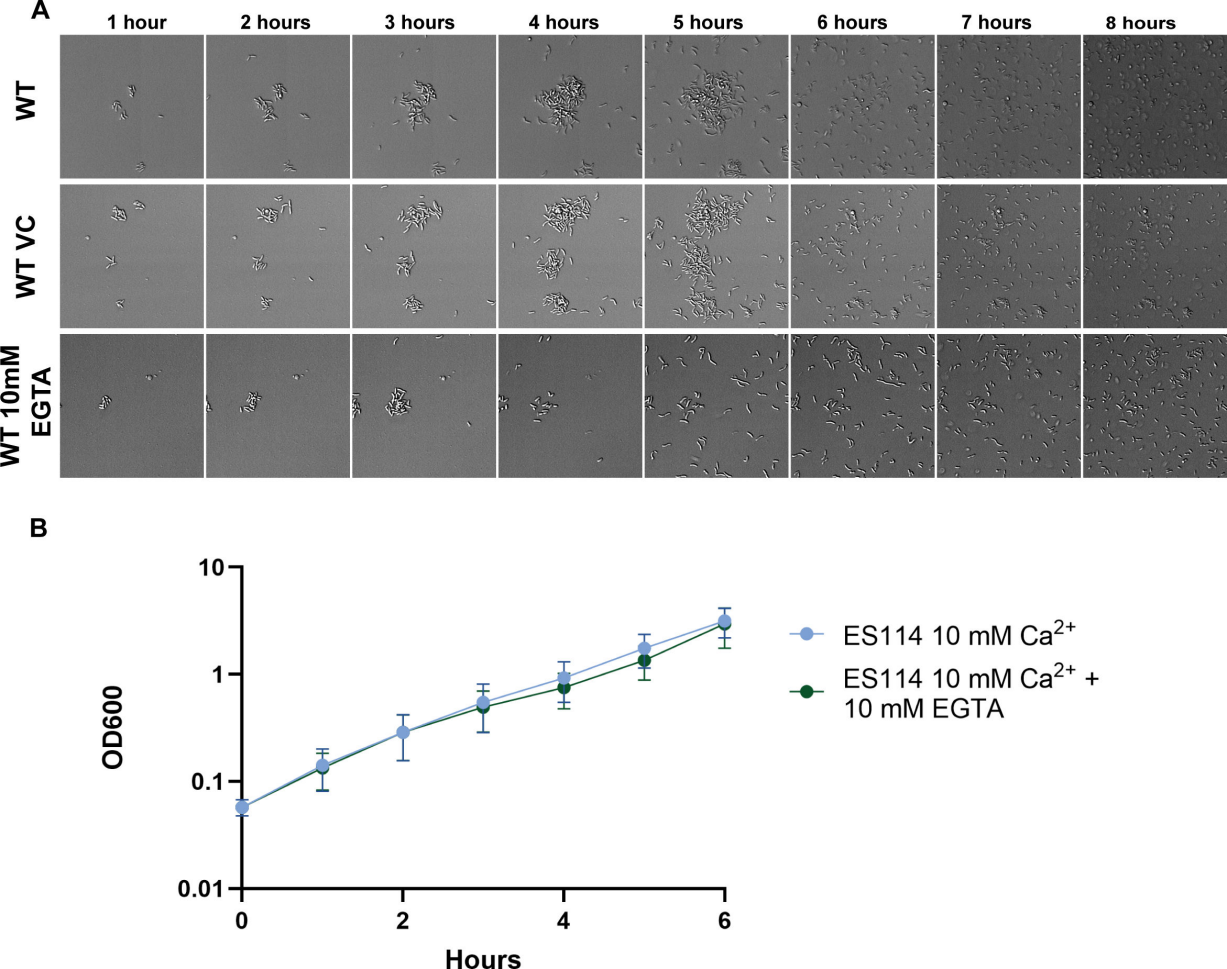
Chelation of free calcium induces dispersal. (**A**) Representative DIC images of 1–8 h of a 10 h experiment comparing ES114 in tTBS with 10 mM added calcium to ES114 in tTBS with 10 mM added calcium that was either given a vehicle control (VC) of 100 µL saltwater (340 mM NaCl) or 100 µL saltwater containing 10 mM EGTA. The EGTA was added immediately after the 3 h time point was captured. (**B**) A 6 h growth curve of ES114 in tTBS conditions with 10 mM added calcium and either no addition or 10 mM EGTA added at time zero. A non-linear regression was fitted to each condition using the exponential growth equation. The regression curves were compared to each other and found to not be significant. Images displayed are 46 µm wide. *n* = 3 biological replicates. The low-contrast vertical banding in panel A is an artifact from the DS-Qi2 CMOS camera.

Our data demonstrated that altering calcium concentrations and exposure times influences cellular attachment, biofilm size, and dispersal timing. Therefore, we hypothesized that levels of free calcium could be part of the signal for *V. fischeri* dispersal under our conditions. We thus decreased the levels of free calcium in the middle of biofilm growth by spiking in the calcium chelator EGTA (Ethyleneglycol- *bis*(β-aminoethyl)-N,N,Nʹ,Nʹ-tetraacetic Acid) during a time-lapse experiment of ES114 in tTBS containing 10 mM calcium. Three hours after the experiment was started, EGTA in salt water was added to a final concentration of 10 mM. After the addition of EGTA, bacteria rapidly dispersed from biofilm structures (Fig. 7A; Movie S7). No further structures were formed after EGTA addition to the wells; however, the bacteria continued to grow in the medium, indicating that the EGTA was not lethal to the bacteria. To further assess the effect of the added EGTA on the viability of *V. fischeri*, we measured the growth of *V. fischeri* with shaking over time. The addition of 10 mM EGTA to a culture did not significantly impact the growth of ES114 (Fig. 7B). These data suggest that the addition of the calcium chelator EGTA at 10 mM is not toxic to *V. fischeri* but does induce dispersal in the time-lapse assay.

### Calcium sensing is required for biofilm attachment and formation

**Fig 8:**
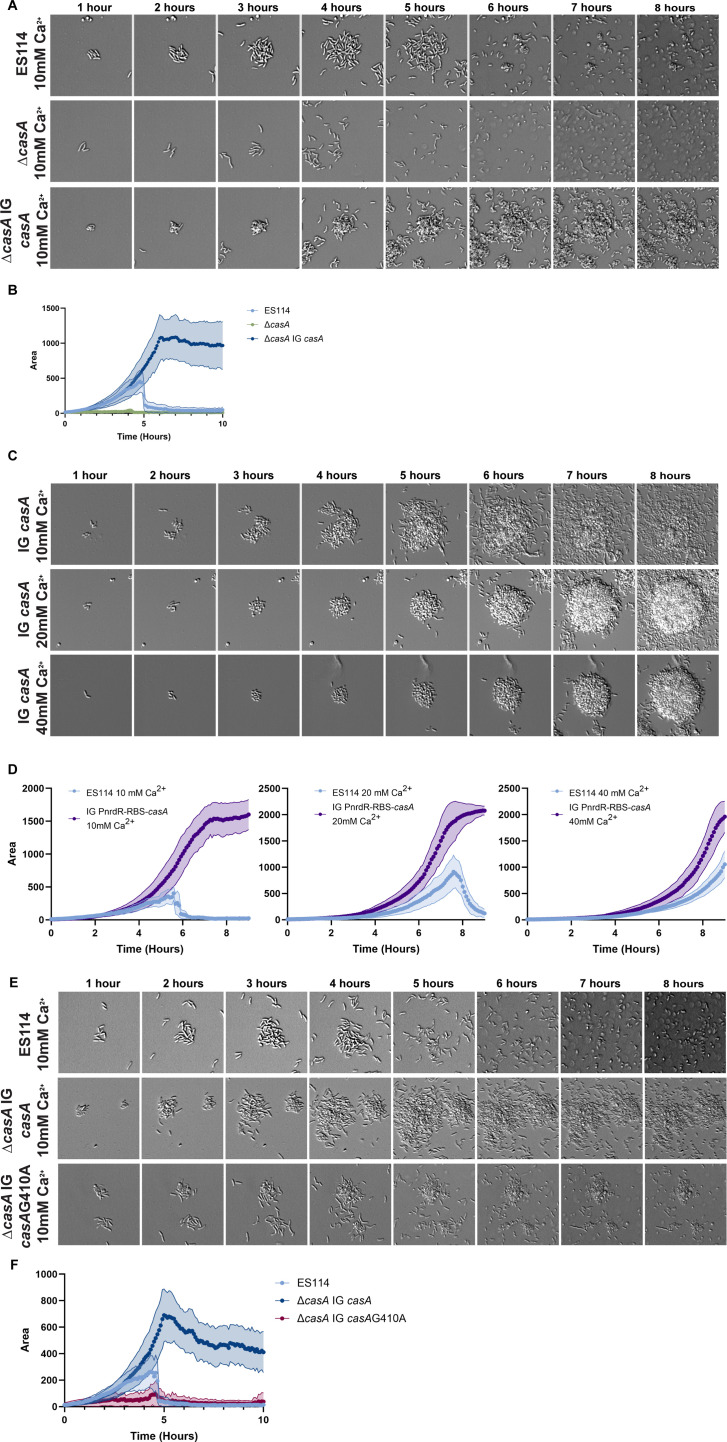
Calcium sensing is required for biofilm attachment and formation. (**A**) Representative DIC images of 1–8 h of a 10 h experiment comparing ES114 with 10 mM calcium to a strain deleted for *casA* (∆*casA*) or a strain complemented for *casA* (∆*casA* IG *casA*). (**B**) Quantification from a representative panel A experiment via ImageJ macro. (**C**) Representative DIC images of 1–8 h of a 10 h experiment comparing a CasA overexpressing strain (IG *casA*) in tTBS media with increasing concentrations of calcium (10 mM, 20 mM, and 40 mM). (**D**) Quantification from a representative panel C experiment via ImageJ macro. (**E**) Representative DIC images of 1–8 h of a 10 h experiment comparing ES114 to a strain complemented for casA (∆*casA* IG *casA*) or a complemented point mutant of *casA* that cannot produce c-di-GMP (∆*casA* IG *casA*-G410A). (**F**) Quantification from a representative panel E experiment via ImageJ macro. Images displayed are 46 microns wide. The images are representative of *n* = 3 biological replicates, and the quantifications include *n* = 15–25 biofilms per condition, ± SD (Shaded).

*V. fischeri* senses calcium, in part, by the transmembrane calcium-responsive DGC CasA (14). Therefore, we asked if CasA was required to promote biofilm formation in the time-lapse assay. Indeed, a *casA* deletion mutant did not readily attach to the surface or form biofilms in tTBS medium containing 10 mM calcium (Fig. 8A; Movie S8; Fig. S6A). Aggregates were limited to 2–8 individual bacteria at most before they detached from the glass surface. These data suggest that calcium sensing in *V. fischeri* is required for stable attachment.

To verify this result, we tested the phenotype of the Δ*casA* mutant that carried a complementation cassette that was generated previously (14) and consisted of a chromosomal copy of *casA* under the control of a non-native promoter and an idealized ribosome binding site (PnrdR-RBS-*casA*). This strain regained the ability to form a biofilm, suggesting that the loss of CasA was indeed responsible for the inability of the Δ*casA* mutant to form a biofilm. However, the complemented strain unexpectedly failed to disperse. Instead, it formed aggregates that increased in area for about 5 h, which plateaued in size around the same time that ES114 dispersed in a coordinated manner (Fig. 8A; Fig. S6A; Movie S8). Given this result and the construction of the complementation cassette, we conclude that this strain likely overexpresses CasA, causing increased activity and, potentially, increased or prolonged sensitivity to calcium, resulting in a dispersal defect.

This result identified a genetic condition that appeared to impair dispersal. To study this phenomenon further, we generated a *casA* overexpression strain in a wild-type background and included the PnrdR-RBS-*casA* cassette (termed “IG *casA*”). This strain phenocopied the Δ*casA* complemented strain for biofilm formation (Fig. 8A and B; Movie S8). We further tested it by examining its response to increasing amounts of calcium (20 and 40 mM) present in the assay and found that it formed larger aggregates that plateaued at later time points and failed to disperse (Fig. 8C and D; Fig. S6B; Movie S8). This result differs from ES114, which disperses at both the 10 mM and 20 mM conditions (Fig. 8D). These data suggest that either sustained or elevated calcium sensing by CasA blocks biofilm dispersal.

Because CasA is a DGC, we hypothesized that this activity, the production of c-di-GMP, was specifically required. We thus assessed the phenotype of a ∆*casA* mutant that expressed a catalytically inactive derivative of *casA* (G410A) (14) under the control of the same non-native promoter and idealized RBS. This strain failed to form the large biofilms of the ∆*casA* IG PnrdR-RBS-*casA* strain, although it did form small aggregates (Fig. 8E and F; Fig. S7; Movie S8). We conclude that calcium is likely detected by CasA, resulting in increased c-di-GMP levels and therefore increased activation of downstream pathways.

## DISCUSSION

In this study, we demonstrated that it is possible to capture the biofilm formation and dispersal process of *V. fischeri* via time-lapse microscopy. We showed that tTBS medium supplemented with 10 mM calcium is a condition sufficient for a productive investigation. We also identified new roles for calcium in controlling both biofilm size and the timing of dispersal. Finally, we found that our assay is broadly applicable to the study of any of a variety of *V. fischeri* strains, as all seven isolates tested formed biofilms under our conditions.

Our time-lapse assay was based on one developed by Bridges and Bassler (20) that we modified for use with *V. fischeri*. In our assay, we employed an area-measurement strategy on individual biofilms captured using differential interference contrast imaging instead of total light attenuation in brightfield. We also adjusted the temperature to 25°C because that temperature promotes *V. fischeri* biofilm formation. In addition, we found that the role of autoinducer between *V. fischeri* and *V. cholerae* differed: in *V. fischeri*, biofilm formation and dispersal patterns did not substantially change when genes for the three autoinducer synthases were deleted, whereas in *V. cholerae,* autoinducers clearly controlled this pathway (20). Of note, the timing of biofilm dispersal by strain ES114 was within about 5–6 h. This is a relatively short dispersal time compared with the 10–12 h timing of *V. cholerae* (34), but it is in line with the known timing of *V. fischeri* biofilm formation and dispersal during squid colonization (35). Our results thus extend the work of Bridges and Bassler (20) by confirming the utility of this imaging-based assay for the discovery of dispersal factors in an additional microbe and by identifying differences both between the two related bacteria and between different *V. fischeri* isolates.

We chose to evaluate biofilm formation and dispersal by representative *V. fischeri* strains that had been differentially assigned into the colonization categories of niche dominant, niche sharing, or ineffectual colonizer (22). We hypothesized, based on previously published data that showed that niche-dominant strains colonize squid hosts faster than the niche-sharing strains (35), that they would form and disperse from biofilms more quickly than the niche-sharing strains under our conditions. However, we could not identify a specific pattern of biofilm formation or dispersal based on niche status. For example, we expected niche-dominant strains MB13B2 and KB2B1 to form a biofilm and disperse quicker than strain ES114 (35), but they did not. Furthermore, MJ11 fails to efficiently colonize *E. scolopes* (11), whereas SR5 can colonize both *Sepiola robusta* and *E. scolopes* (36), but both strains were capable of biofilm formation and dispersal in our time-lapse assay. Of note, both strains lack the sensor kinase RscS, an important regulator of biofilm formation and critical for colonization in many *V. fischeri* strains (11, 37), but this absence did not prevent biofilm formation in this *in vitro* assay. Together, these data indicate that neither colonization differences nor the lack of an important regulator are predictive of dynamics in our *in vitro* assay. This lack of predictive power suggests that our conditions insufficiently match those involved in early colonization steps and that further development of our assay is necessary to observe correlations with colonization. However, there were notable differences (e.g., timing and biofilm sizes) among the strains that are worthy of further investigation.

One surprising result was that neither individual mutants for SYP, cellulose, or Lap nor the triple mutant had biofilm formation or dispersal defects. We expected that deletion of one, and especially all three, would result in lower biofilm formation or attachment. However, our data clearly show these mutants were capable of attachment and biofilm formation. These experiments suggest that there must be one or more other surface components responsible for attachment and initial biofilm formation. Potential components are flagella, LPS molecules, pili, curli, and other polysaccharide(s). In other *Vibrio* species, it has been shown that flagella, although important for motility, also play a role in early biofilm formation by interacting with glass surfaces (38, 39). *V. fischeri* has multiple sheathed (encased in membrane) flagella localized to a single pole of the cell (40). Although some roles have been attributed to the sheathed flagella, many aspects remain unknown, and it is quite possible that a component of the sheath, such as LPS, is required for initial attachment. *V. fischeri* encodes a locus for curli along with nine putative pilus loci, but most remain unstudied (41–43). The potentially redundant nature of the 10 pili loci, including curli, would require the construction of many mutants to determine if pili play a role. Finally, there are other polysaccharides and/or other polysaccharide loci, including *VF_1057-VF0180* (44) and the *vps*-like locus *VF_0352-VF_0344* (45, 46), that could promote binding. The targets described above are only some of the potential candidates, but investigation of these potential factors could lead to a better understanding of the early biofilm formation process.

Calcium was a key factor in successful attachment and aggregate formation in the time-lapse assay. For both the wild-type and *casA* overexpression strains, increased calcium concentrations changed the timing, duration, and size of biofilms. Increased calcium also changed biofilm appearance: when calcium was present at levels of 20 mM or above, the strains produced biofilms with a more uniform round shape that seemed to be taller (a characteristic not captured in our studies). These biofilms more closely resemble the classic mushroom-shaped biofilms reported in other species (47). Our results suggest that because *V. fischeri* is equipped to sense and respond to higher levels of calcium, a niche exists where these bacteria encounter these conditions.

The calcium-sensitive DGC, CasA, was required for stable attachment and biofilm formation. Loss of CasA or disruption of its DGC activity is known to prevent the calcium-induced increase in c-di-GMP levels (14). Given these data and our results with the CasA overproduction strain, we hypothesize that high levels of CasA lead to increased calcium sensitivity and therefore increased c-di-GMP production, which in turn promotes and extends biofilm formation. Correspondingly, the wild-type strain, which both forms and disperses from biofilms, may generate c-di-GMP in response to calcium, then stop making it and/or degrade it. Lower c-di-GMP levels could be achieved in multiple ways, such as through lowered transcription of *casA*, degradation of CasA, or an antagonistic PDE. We hypothesize that whatever the mechanism, it involves specific timing to lower the pool of available c-di-GMP to turn off the pathways activated by CasA and trigger dispersal. Of note, we found that disruption of the enzymatic GGDEF motif did not result in a full null phenotype in our assay; rather than failing to productively attach like its Δ*casA* parent, the CasA-G410A expressing strain attached and formed very small biofilms. These data indicate that CasA-G410A may retain some activity, such as c-di-GMP binding via its RxxD motif (48) or through protein-protein interactions. However, the G410A-overexpression strain failed to form the large biofilms of the CasA-overexpression strain, indicating that diguanylate cyclase activity is critical for that phenotype.

The buildup of c-di-GMP mediated by CasA is thought to activate the transcription factor VpsR which, in turn, activates or represses gene transcription (14). One gene cluster upregulated by VpsR is the *bcs* locus, which is responsible for cellulose production (8, 14). However, based on the lack of a phenotype for the Δ*bcsA* mutant, we conclude that cellulose alone is not responsible for the increased biofilm formation mediated by CasA. These data indicate that the c-di-GMP produced by CasA regulates cell processes via another pathway or that VpsR has another target(s) besides *bcs* that is important for biofilm formation (49).

In summary, we demonstrated that *V. fischeri* biofilm formation and dispersal relies on calcium sensing via CasA and c-di-GMP production in a short-term biofilm formation and dispersal assay. The experimental conditions we tested allowed us to delineate environmental and genetic factors that impact bacterial cell attachment, biofilm accumulation, and dispersal, with the potential to examine these events across multiple strains of *V. fischeri*. Finally, we determined that the early biofilm is independent of already established biofilm components (SYP, Cellulose, and LapV), leaving exciting new directions to explore to understand how *V. fischeri* makes contact with surfaces and undergoes early cell-cell interactions.

## MATERIALS AND METHODS

### Strains and media

*V. fischeri* strains, plasmids, and primers used in this study are listed in Tables 1 to 3. ES114 was the parent strain for all genetically manipulated strains. *E. coli* strains were grown in lysogeny broth (LB) (1% tryptone, 0.5% yeast extract, and 1% sodium chloride) (50). For genetic manipulation and routine passaging, *V. fischeri* strains were grown in Luria-Bertani salt (LBS) medium (1% tryptone, 0.5% yeast extract, 2% sodium chloride, and 50 mM Tris, pH 7.5) (51, 52). LB and LBS were solidified with 1.5% agar (final) as appropriate. For imaging experiments, *V. fischeri* strains were grown in Tris-buffered tryptone-based (tTBS) medium (1% tryptone, 2% sodium chloride, and 50 mM Tris, pH 7.5) (21). tTBS medium was supplemented with 10 mM, 20 mM, or 40 mM CaCl_2_ where noted. For *tfoX*-mediated transformation, Tris-minimal medium (TMM) (300 mM NaCl, 0.1% ammonium chloride, 10 mM *N-*acetylglucosamine, 50 mM MgSO_4_, 10 mM KCl, 10 mM CaCl_2_, 0.0058% K_2_HPO_4_, 10 µM ferrous ammonium sulfate, and 100 mM Tris, pH 7.5) was used (53). For *V. fischeri,* antibiotics were used as follows: erythromycin (Erm), 2.5 µg/mL; trimethoprim (Trim), 10 µg/mL; kanamycin (Kan), 100 µg/mL; and chloramphenicol (Cm), 5 µg/mL. For *E. coli*, antibiotics were used as follows: cm, 12.5 µg/mL; Kan, 50 µg/mL. Thymidine (Thy) was added to a concentration of 0.3 mM for growth of the thymidine auxotroph strain π3813 (54) that carries the conjugal plasmid pEVS104 (55) used to facilitate conjugations and the flippase plasmid pKV496 (56).

**TABLE 1:** Strains used in this study^*a*^^,^^*b*^

Strain	Genotype	Construction	Reference
CL39	*luxS*::Kan	N/A	(57)
ES114	Sharing isolate from *E. scolopes*	N/A	(58)
ES213	Dominant isolate from *E. scolopes*	N/A	(58)
JE126	∆*bcsA*::FRT-Trim^R^ Δ*sypQ*::FRT-Cm^R^ Δ*lapV*-1500::FRT-Erm^R^	TT KV8753 with gKV8613	This study
JE176	∆*bcsA*::FRT Δ*sypQ*::FRT Δ*lapV*-1500::FRT	Erm^S^ derivative of JE126 made using pKV496	This study
JE192	*luxI* frameshift ∆*ainS*::FRT-Erm^R^	TT VCW2G7 with gKV9367	This study
JE193	*luxI* frameshift Δ*ainS*::FRT-Erm^R^ *luxS*::Kan^R^	TT JE192 with gCL39	This study
JE224	IG::P*nrdR*-RBS-*casA*-HA	TT ES114 with gKV9821	This study
KB2B1	Dominant isolate from *E. scolopes*	N/A	(27)
KV8191	∆*sypQ*::FRT-Cm^R^	N/A	(56)
KV8232	IG::Erm^R^-trunc Trim^R^	N/A	(56)
KV8613	∆*lapV-1500*::FRT-Erm^R^	N/A	(7)
KV8616	∆*bcsA*::FRT-Trim^R^	N/A	(7)
KV8735	∆*lapG*::FRT-Erm^R^	N/A	(7)
KV8753	∆*bcsA*::FRT-Trim^R^ Δ*sypQ*::FRT-Cm^R^	N/A	(7)
KV8920	∆*casA*::FRT-Spec^R^	N/A	(14)
KV9367	∆*ainS*::FRT-Erm^R^	N/A	(59)
KV9821	∆*casA*::FRT-Spec^R^ IG::P*nrdR*-RBS-*casA*-HA	N/A	(14)
KV9822	∆*casA*::FRT-Spec^R^ IG::P*nrdR*-RBS-*casA-G410A*-HA	N/A	(14)
MB13B2	Dominant isolate from *E. scolopes*	N/A	(27)
MB14A5	Sharing isolate from *E. scolopes*	N/A	(27)
MJ11	Isolate from *M. japonica*, colonization-defective in *E. scolopes*	N/A	(42)
SR5	Isolate from *S. robusta*	N/A	(60)
VCW2G7	*luxI* frameshift	N/A	(61)

^*a*^ Abbreviations: FRT, Flp recombinase target; HA, HA epitope-tagged; IG, intragenic region between genes *yeiR* and *glmS*; N/A, not applicable because construction of the indicated strain was constructed reported previously.

^*b*^ Derivation of strains constructed in this study: TT, TfoX-mediated Transformation with a strain containing the *tfoX* gene on a plasmid.

**TABLE 2:** Plasmids used in this study

Name	Description	Reference
pEVS104	Conjugal plasmid	(55)
pJJC4	*tfoX^+^ litR ^+^* + Cm^R^	(62)
pKV496	*flp^+^* + Kan^R^	(56)
pLosTfox	*tfoX*^+^ + Cm^R^	(63)
pLosTfox-Kan	*tfoX*^+^ + Kan^R^	(64)

**TABLE 3:** Primers used in this study

Primer number	Sequence^*a*^
181	aaaaaagaattcAGGATAGATAATAGCGAATAGG
443	CGGTAATACTCCATAAGTTCTTTCAC
1188	GGTAATGCTGGGCGACTAG
1252	ACTTCCACTTGCCGCTATAG
1253	GAATCTTATCGCTTGATGCTTG
1258	GTGTACATAGCTCACCTTCAG
1259	GCAATGGTTGAGATCATGTAAA
1297	ATGGTTGGGCTAAGAGATGGC
1318	ATGTAATCGTACTTTGAGAGCTG
1487	GGTCGTGGGGAGTTTTATCC
2034	CGGTATATAGGAAATTTACAATCTG
2290	AAGAAACCGATACCGTTTACG
2442	TCGCTTGCTTCTACTTCTTTACCTTCTAGTT
2443	CCATTACCATCATCGACAACAATCTCAAAG

^*a*^ Lowercase letters indicate nonbinding or tail sequences.

### Strain construction

Mutations in *V. fischeri* ES114 were generated through TfoX-mediated transformation as described previously (53, 56, 62–64). gDNA was transformed into recipient *V. fischeri* carrying a TfoX-overexpression plasmid pJJC4 (62), and recombinant cells were selected by plating on media containing appropriate antibiotics. Strains were streaked without selection to ensure the TfoX-overexpressing plasmid was lost prior to performing experiments. All genetic manipulations were confirmed by PCR with outside primers using Promega *Taq* polymerase.

### Time-lapse imaging

Bacteria were prepared from overnight cultures inoculated from single colonies into tTBS and grown overnight at 24°C. Subcultures were started the next day in the same conditions, initially normalized to an optical density of 0.5 at 600 nM (OD_600_), then grown for seven hours. The cultures were diluted 1:200,000, then seeded into 8-well chambered cover glass (borosilicate) slides (Ibidi) for 1 h in tTBS medium containing or lacking 10 mM CaCl_2_. Planktonic bacteria were removed by three washes with 500 µL tTBS medium, and a final volume of 500 µL tTBS medium, containing 10 mM CaCl_2_ or higher levels as noted, was added to each chamber. Time-lapse fields were configured immediately, and imaging began 45 min after the medium change. Images were captured on a Nikon Ti2-E inverted microscope using differential interference contrast. The system was equipped with Nikon perfect focus, an Okolab stage-top incubation chamber held at 25°C, a DS-Qi2 CMOS camera, and a CFI Plan Apochromat Lambda D 40X air NA 0.95 objective. Five fields per well were imaged every 5 min for 10 h. Movies assembled from these images contain all the time points.

### Biofilm size measurements

Time-lapse images were computationally analyzed using a custom macro written for the FIJI package of Image J (28). The code for measuring individual biofilms is available in a GitHub repository: [https://github.com/abbykroken/v_fischeri_biofilm/]. In brief, the user is prompted to draw a region of interest (ROI) around a single biofilm; we chose biofilms that did not merge with a neighboring biofilm during the time frame of analysis. The image is registered to account for drift using the Correct 3D Drift plugin (65). Bacteria observed in differential interference contrast are thresholded first by using a Sobel edge detector (“Find Edges”). Within the ROI, isolated planktonic bacteria are removed using a size filter. The largest object (which corresponds to the biofilm during its growth phase) is retained. Its area is calculated for each frame, which is reported as a results table. A color-coded overlay is generated for the user to visually check for accuracy. Loss of the large object corresponds visually to bacterial dispersal.

For each experimental condition, the experiment was performed at least three times on different days. The absolute time of dispersal ranged from 300 min to 360 min for ES114. Thus, the figures show a single experiment representative of three biological replicates, and ES114 was always included as a comparator to observe reproducible trends. Error bars represent variability between 15 and 25 individual biofilms within a biological replicate. We did not further pursue statistical measurements as the observed patterns were striking and generally followed three trends: absence of biofilm, normal biofilm, and dispersal, or biofilm with delayed/absent dispersal. In Fig. 6C/D&8C/D, we only quantified until hour 9 because at hour 10, there were surrounding planktonic and adhered bacteria that complicated the analyses. Supplemental figures show the variability in each experiment. Movies were assembled using Adobe Premiere.

### Time-lapse with EGTA addition

For the EGTA addition experiments, the approach was the same as that described above, except as follows. Three hours after imaging was started, the coverslip was carefully removed, and 100 μL of either vehicle control (VC) of 340 mM salt water or the same saltwater solution containing 60 mM EGTA was gently added to appropriate wells to achieve a final concentration of 10 mM EGTA in a final volume of 600 μL. Our addition of the relatively large volume of 100 μL was designed to facilitate rapid diffusion throughout the sample.

### Bacterial growth curves

Single colonies were inoculated into 5 mL of tTBS medium, and the cultures were grown overnight at 24°C with shaking. The next morning, the strains were normalized to an OD_600_ of 0.05 in 30 mL of tTBS in 250 mL baffled flasks, then grown at 24°C with shaking. Calcium was added to a final concentration of 10 mM in each flask, and EGTA was added to 10 mM (final) in indicated flasks prior to inoculation with bacteria. Aliquots of 1 mL were taken hourly for OD_600_ measurements with a spectrophotometer. To maintain accuracy, samples were diluted for measurement when they reached an OD_600_ above 1.0. The graphs were created and statistics were performed with Prism 9 (GraphPad, San Diego, CA, USA). Non-linear regression lines were fit to individual growth curves using exponential growth equations. Curves for each condition were then compared with each other for significant differences.

## SUPPLEMENTAL MATERIAL

Supplemental figures jb.00077-25-s0001.pdf Figures S1 to S7.

Supplemental movie legends jb.00077-25-s0002.pdf Legends for the Movies S1 to S8.

Movie S1 jb.00077-25-s0003.mp4 Wild type squid and fish isolates have diverse biofilm phenotypes. Representative DIC time-lapse videos of a 10-hour experiment with images taken every 5 minutes. Experiment performed in tTBS containing 10 mM CaCl2 at 25°C. n = 3 biological replicates.

Movie S2 jb.00077-25-s0004.mp4 Early aggregation and attachment are independent of SYP, cellulose, and LapV. Representative DIC time-lapse videos of a 10-hour experiment for ES114 and mutants defective for SYP (∆sypQ), cellulose (∆bcsA), or LapV surface adhesin (∆lapV-1500), or a triple mutant deficient for all three components (∆bcsA ΔsypQ ΔlapV-1500). n = 3 biological replicates.

Movie S3 jb.00077-25-s0005.mp4 *V. fischeri* dispersal from early biofilms is largely independent of lapG. Representative DIC time-lapse videos of a 10-hour experiment comparing ES114 to a LapG protease deficient strain (∆lapG). n = 3 biological replicates.

Movie S4 jb.00077-25-s0006.mp4 Coordinated dispersal is independent of autoinducer signaling. Representative DIC time-lapse videos of a 10-hour experiment comparing ES114 to a strain deficient for all three autoinducers (luxI-frameshift ∆ainS luxS). n = 3 biological replicates.

Movie S5 jb.00077-25-s0007.mp4 Calcium is required for aggregate formation. Representative DIC time-lapse video of a 10-hour experiment comparing ES114 in tTBS media with 10 mM added calcium to ES114 in tTBS without added calcium. n = 3 biological replicates.

Movie S6 jb.00077-25-s0008.mp4 Calcium time of addition and concentration. Representative DIC time-lapse videos of a 10-hour experiment comparing ES114 with 10 mM calcium added just prior to imaging, ES114 that was exposed to 10 mM calcium in the 1-hour incubation period, and ES114 in three calcium concentrations (10 mM, 20 mM, 40 mM). n = 3 biological replicates.

Movie S7 jb.00077-25-s0009.mp4 Chelation of free calcium induces dispersal. Representative DIC time-lapse videos of a 10-hour experiment comparing ES114 in tTBS with 10 mM added calcium to ES114 in tTBS with 10 mM added calcium that was either given a vehicle control (VC) of 100 uL saltwater (340 mM NaCl) or 100 uL saltwater containing 10 mM EGTA. n = 3 biological replicates.

Movie S8 jb.00077-25-s0010.mp4 Calcium sensing is required for biofilm attachment and formation. Representative DIC time-lapse videos of a 10-hour experiment comparing ES114 with 10 mM calcium to a strain deleted for casA (∆casA), a ΔcasA mutant complemented with either casA (∆casA IG casA) or a derivative casA that cannot produce c-di-GMP (∆casA IG casA-G410A), and a CasA overexpressing strain (IG casA) in tTBS media with increasing concentrations of calcium (10 mM, 20 mM, 40 mM). n = 3 biological replicates.

ASM does not own the copyrights to Supplemental Material that may be linked to, or accessed through, an article. The authors have granted ASM a non-exclusive, world-wide license to publish the Supplemental Material files. Please contact the corresponding author directly for reuse.
