# Supplementary material for: Calcium signaling controls early stage biofilm formation and dispersal in Vibrio fischeri
Source: J Bacteriol. 2025 May 14;207(6):e00077-25. doi: 10.1128/jb.00077-25 (PMC12186489; doi:10.1128/jb.00077-25)
Supplement: Supplemental figures — Figures S1 to S7. [file jb.00077-25-s0001.pdf]

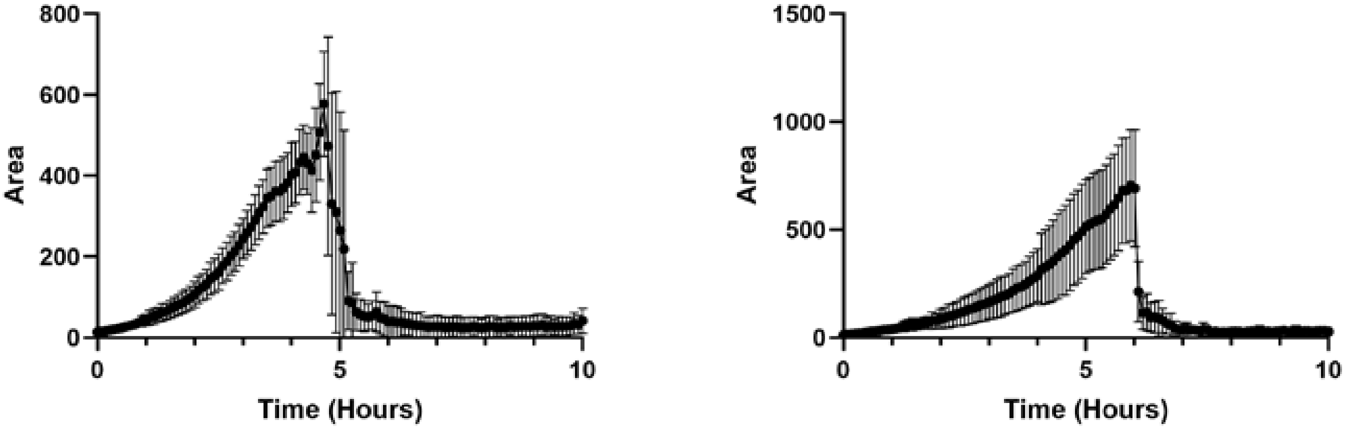

Supplemental Figure 1. Day-to-day variation in ES114 dispersal timing. Biological replicates of time lapse experiments quantified via ImageJ are shown for ES114 on different experiment days. Colors denote day of experiment. n = 3 biological replicates and n = 15-25 biofilms per condition, ± SD.

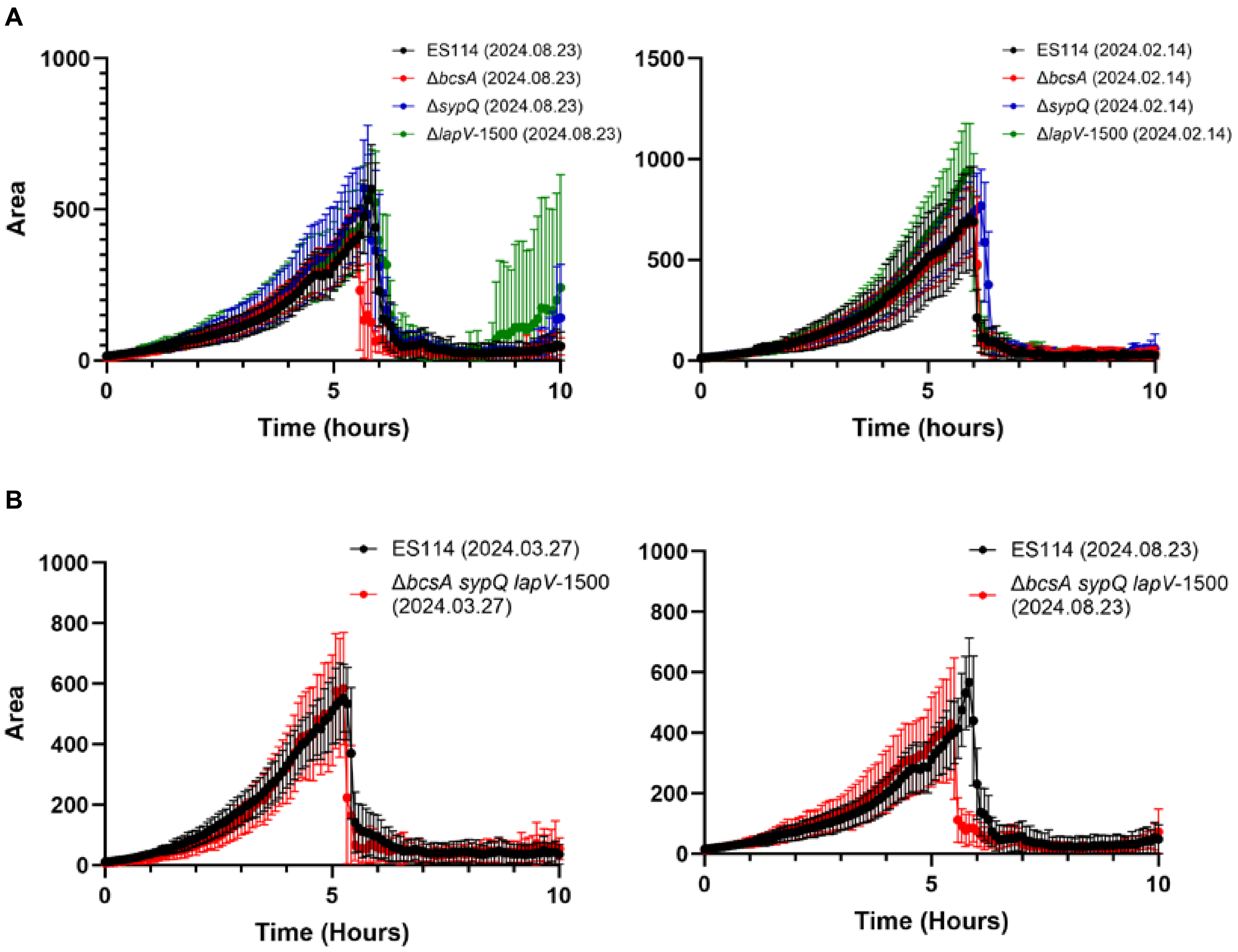

Supplemental Figure 2. Early aggregation and attachment are independent of known biofilm components. Biological replicates of time lapse experiments quantified via ImageJ are shown for (A) ES114 and mutants defective for cellulose (ΔbcsA), SYP (ΔsypQ), lap surface adhesin (ΔlapV-1500), or a (B) triple mutant deficient for each component (ΔbcsA ΔsypQ Δ lapV-1500). n = 3 biological replicates and n = 15-25 biofilms per condition, ± SD.

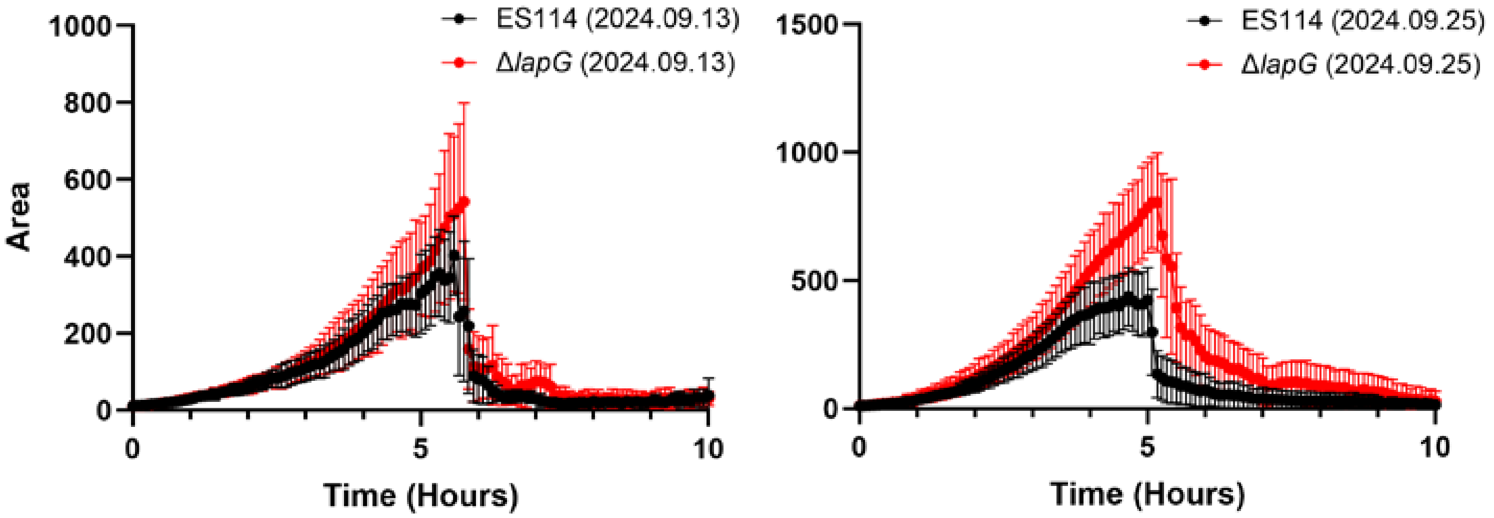

Supplemental Figure 3. V. fischeri dispersal from early biofilms is largely independent of the lap system. Biological replicates of time lapse experiments quantified via ImageJ are shown for ES114 compared to a LapG protease deficient strain (ΔlapG). n = 3 biological replicates and n = 15-25 biofilms analyzed per condition, ± SD.

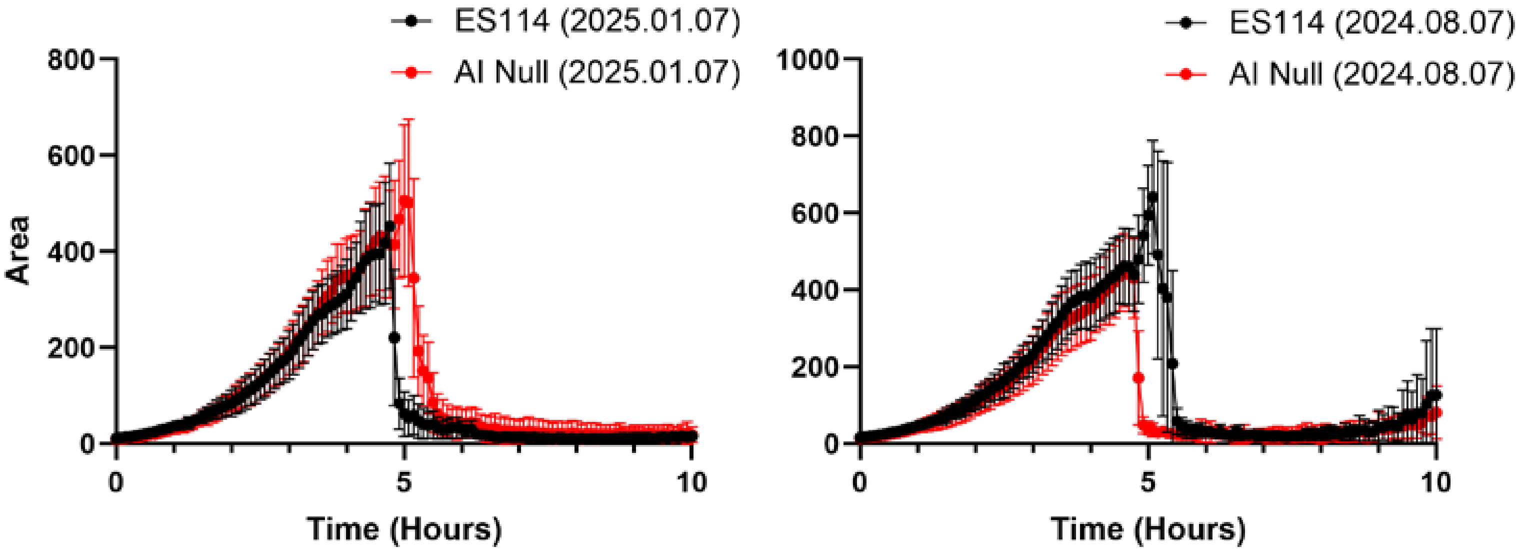

Supplemental Figure 4. Coordinated dispersal is independent of autoinducer signaling. Biological replicates of time lapse experiments quantified via ImageJ are shown for ES114 compared to a strain deficient for all three autoinducers (luxI-frameshift ΔainS luxS). n = 3 biological replicates and n = 15-25 biofilms analyzed per condition, ± SD.

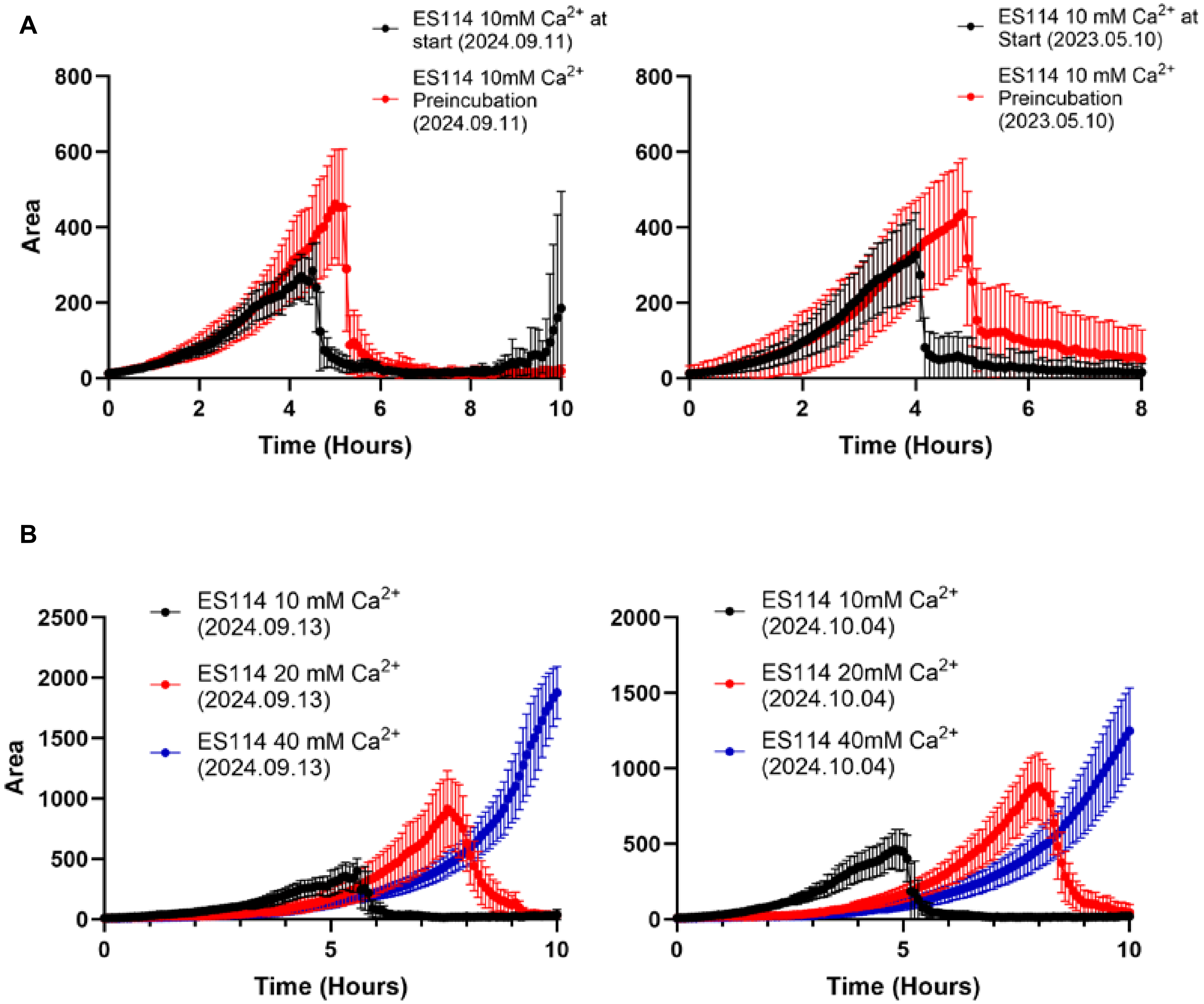

Supplemental Figure 5. Calcium time of addition and concentration. Biological replicates of time lapse experiments quantified via ImageJ are shown for ES114 with 10 mM calcium added just prior imaging and ES114 that was exposed to 10 mM calcium in the 1-hour incubation period. (B) Biological replicates of time lapse experiments quantified via ImageJ are shown for ES114 in three calcium concentrations (10 mM, 20 mM, 40 mM). n = 3 biological replicates and n = 10-25 biofilms analyzed per condition, ± SD.

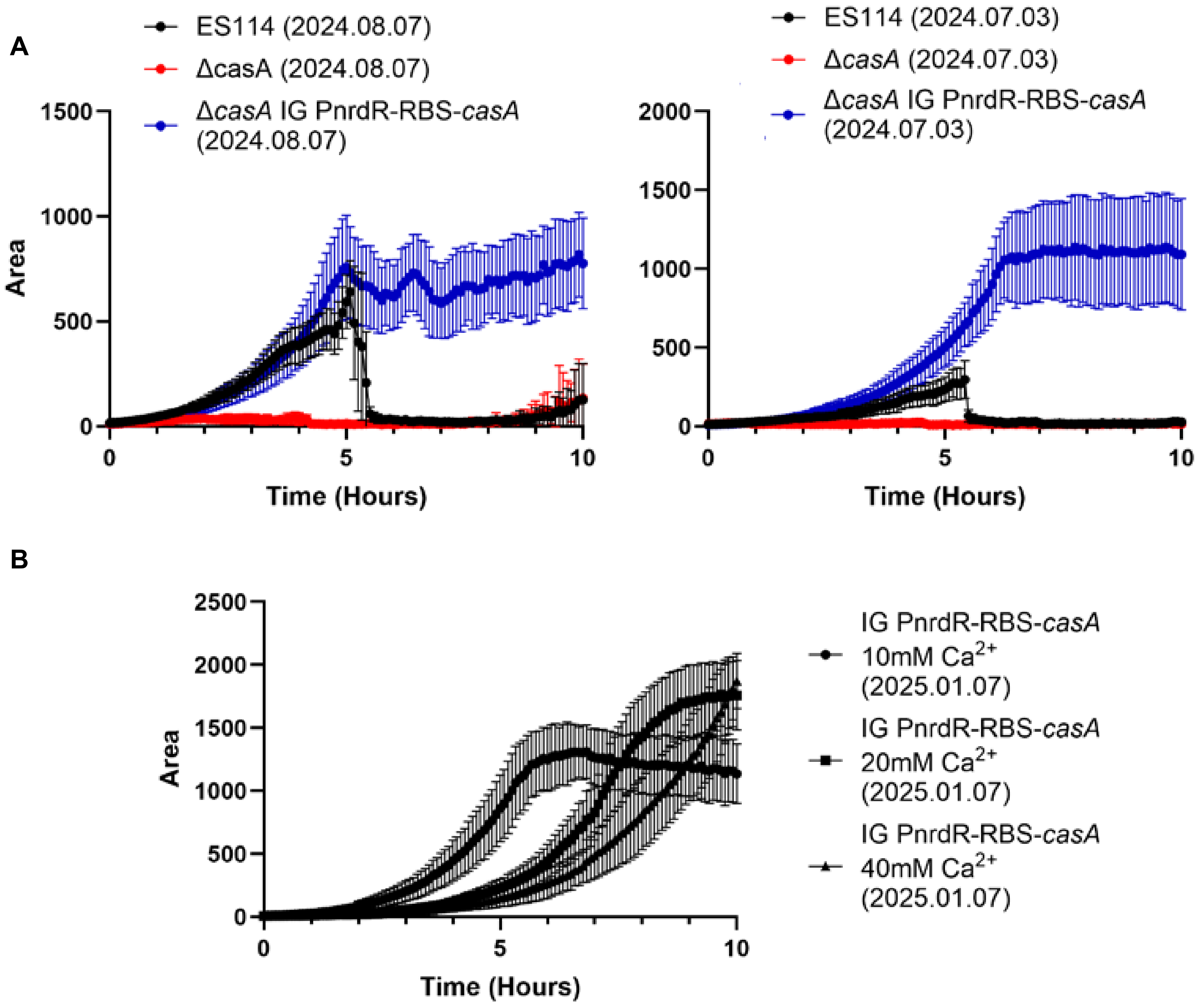

Supplemental Figure 6. Calcium sensing is required for biofilm attachment and formation. Biological replicates of time lapse experiments quantified via ImageJ are shown comparing (A) ES114 with 10 mM calcium to a strain deleted for casA (ΔcasA) or a strain complemented for casA (ΔcasA IG casA). (B) Biological replicates of time lapse experiments quantified via ImageJ are shown comparing a CasA overexpressing strain (IG casA) in tTBS media with increasing concentrations of calcium (10 mM, 20 mM, 40 mM). n = 3 biological replicates and n = 10-25 biofilms analyzed per condition, ± SD.

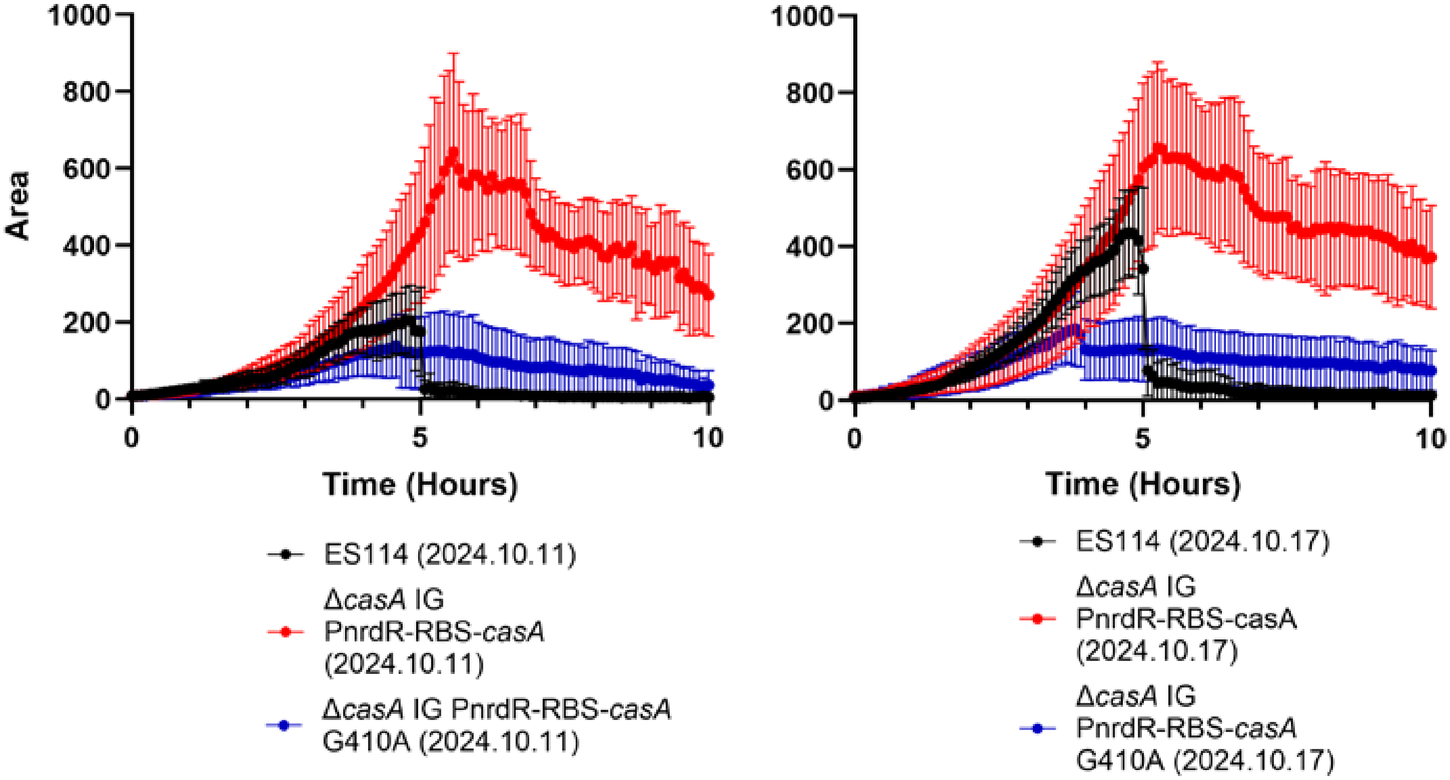

Supplemental Figure 7. Cyclic-di-GMP production of CasA is required for productive biofilm formation. Biological replicates of time lapse experiments quantified via ImageJ are shown comparing (A) ES114 with 10 mM calcium to a strain deleted for casA (ΔcasA) or complemented point mutant of casA that cannot produce c-di-GMP (ΔcasA IG casA-G410A). n = 3 biological replicates and n = 15-25 biofilms analyzed per condition, ± SD.
