# Supplementary material for: Calcium signaling controls early stage biofilm formation and dispersal in Vibrio fischeri
Source: J Bacteriol. 2025 May 14;207(6):e00077-25. doi: 10.1128/jb.00077-25 (PMC12186489; doi:10.1128/jb.00077-25)
Supplement: Supplemental movie legends — Legends for the Movies S1 to S8. [file jb.00077-25-s0002.pdf]

Supplemental Figure Legends

Supplemental Video 1. Wild type squid and fish isolates have diverse biofilm phenotypes. Representative DIC time-lapse videos of a 10-hour experiment with images taken every 5 minutes. Experiment performed in tTBS containing 10 mM $CaCl_2$ at 25°C. *n* = 3 biological replicates.

Supplemental video 2. Early aggregation and attachment are independent of SYP, cellulose, and LapV. Representative DIC time-lapse videos of a 10-hour experiment for ES114 and mutants defective for SYP (Δ*sypQ*), cellulose (Δ*bcsA*), or LapV surface adhesin (Δ*lapV-1500*), or a triple mutant deficient for all three components (Δ*bcsA* Δ*sypQ* Δ*lapV-1500*). *n* = 3 biological replicates.

Supplemental Video 3. *V. fischeri* dispersal from early biofilms is largely independent of *lapG*. Representative DIC time-lapse videos of a 10-hour experiment comparing ES114 to a LapG protease deficient strain (Δ*lapG*). *n* = 3 biological replicates.

Supplemental Video 4. Coordinated dispersal is independent of autoinducer signaling. Representative DIC time-lapse videos of a 10-hour experiment comparing ES114 to a strain deficient for all three autoinducers (*luxI*-frameshift Δ*ainS luxS*). *n* = 3 biological replicates.

Supplemental Video 5. Calcium required for aggregate formation. Representative DIC time-lapse video of a 10-hour experiment comparing ES114 in tTBS media with 10 mM added calcium to ES114 in tTBS without added calcium. *n* = 3 biological replicates.

Supplemental Video 6. Calcium time of addition and concentration. Representative DIC time-lapse videos of a 10-hour experiment comparing ES114 with 10 mM calcium added just prior imaging, ES114 that was exposed to 10 mM calcium in the 1-hour incubation period, and ES114 in three calcium concentrations (10 mM, 20 mM, 40 mM). *n* = 3 biological replicates.

Supplemental Video 7. Chelation of free calcium induces dispersal. Representative DIC time-lapse videos of a 10-hour experiment comparing ES114in tTBS with 10 mM added calcium to ES114 in tTBS with 10 mM added calcium that was either given a vehicle control (VC) of 100 uL saltwater (340 mM NaCl) or 100 uL saltwater containing 10 mM EGTA. *n* = 3 biological replicates.

Supplemental Video 8. Calcium sensing is required for biofilm attachment and formation. Representative DIC time-lapse videos of a 10-hour experiment comparing ES114 with 10 mM calcium to a strain deleted for *casA* (Δ*casA*), a Δ*casA* mutant complemented with either *casA* (Δ*casA* IG *casA*) or a derivative *casA* that cannot produce c-di-GMP (Δ*casA* IG casA-G410A), and a CasA overexpressing strain (IG *casA*) in tTBS media with increasing concentrations of calcium (10 mM, 20 mM, 40 mM). *n* = 3 biological replicates.

Supplemental Figure 1. Day-to-day variation in ES114 dispersal timing. Biological replicates of time lapse experiments quantified via ImageJ are shown for ES114 on different experiment days. Colors denote day of experiment. *n* = 3 biological replicates and *n* = 15-25 biofilms per condition, ± SD.

Supplemental Figure 2. Early aggregation and attachment are independent of known biofilm components. Biological replicates of time lapse experiments quantified via ImageJ are shown for

(A) ES114 and mutants defective for cellulose (Δ*bcsA*), SYP (Δ*sypQ*), *lap* surface adhesin (Δ*lapV-1500*), or a (B) triple mutant deficient for each component (Δ*bcsA* Δ*sypQ* Δ*lapV-1500*). *n* = 3 biological replicates and *n* = 15-25 biofilms per condition, ± SD.

Supplemental Figure 3. *V. fischeri* dispersal from early biofilms is largely independent of *lapG*. Biological replicates of time lapse experiments quantified via ImageJ are shown for ES114 compared to a LapG protease deficient strain (Δ*lapG*). *n* = 3 biological replicates and *n* = 15-25 biofilms analyzed per condition, ± SD.

Supplemental Figure 4. Coordinated dispersal is independent of autoinducer signaling. Biological replicates of time lapse experiments quantified via ImageJ are shown for ES114 compared to a strain deficient for all three autoinducers (*luxI*-frameshift Δ*ainS luxS*). *n* = 3 biological replicates and *n* = 15-25 biofilms analyzed per condition, ± SD.

Supplemental Figure 5. Calcium time of addition and concentration. Biological replicates of time lapse experiments quantified via ImageJ are shown for ES114 with 10 mM calcium added just prior imaging and ES114 that was exposed to 10 mM calcium in the 1-hour incubation period. (B) Biological replicates of time lapse experiments quantified via ImageJ are shown for ES114 in three calcium concentrations (10 mM, 20 mM, 40 mM). *n* = 3 biological replicates and *n* = 10-25 biofilms analyzed per condition, ± SD.

Supplemental Figure 6. Calcium sensing is required for biofilm attachment and formation. Biological replicates of time lapse experiments quantified via ImageJ are shown comparing (A) ES114 with 10 mM calcium to a strain deleted for *casA* (Δ*casA*) or a strain complemented for *casA* (Δ*casA* IG *casA*). (B) Biological replicates of time lapse experiments quantified via ImageJ are shown comparing a CasA overexpressing strain (IG *casA*) in tTBS media with increasing concentrations of calcium (10 mM, 20 mM, 40 mM). *n* = 3 biological replicates and *n* = 10-25 biofilms analyzed per condition, ± SD.

Supplemental Figure 7. Cyclic-di-GMP production of CasA is required for productive biofilm formation. Biological replicates of time lapse experiments quantified via ImageJ are shown comparing (A) ES114 with 10 mM calcium to a strain deleted for *casA* (Δ*casA*) or complemented point mutant of *casA* that cannot produce c-di-GMP (Δ*casA* IG *casA*-G410A). *n* = 3 biological replicates and *n* = 15-25 biofilms analyzed per condition, ± SD.
